# Machine learning approaches for image classification in developmental biology and clinical embryology

**DOI:** 10.1242/dev.202066

**Published:** 2025-02-17

**Authors:** Camilla Mapstone, Berenika Plusa

**Affiliations:** Faculty of Biology, Medicine and Health (FBMH), Division of Developmental Biology & Medicine, Michael Smith Building, Oxford Road, University of Manchester, Manchester M13 9PT, UK

**Keywords:** IVF, Machine learning, Image analysis, Medical data analysis

## Abstract

The rapid increase in the amount of available biological data together with increasing computational power and innovative new machine learning algorithms has resulted in great potential for machine learning approaches to revolutionise image analysis in developmental biology and clinical embryology. In this Spotlight, we provide an introduction to machine learning for developmental biologists interested in incorporating machine learning techniques into their research. We give an overview of essential machine learning concepts and models and describe a few recent examples of how these techniques can be used in developmental biology. We also briefly discuss latest advancements in the field and how it might develop in the future.

## Introduction

The amount of available biological data has grown exponentially in recent years, and with this comes the challenge of analysing and extracting knowledge from these datasets. Manual analysis can be subjective, time-consuming and, in some cases, completely infeasible, so there is a huge potential for machine learning (ML) techniques to revolutionise biological research in all areas, from basic research to clinical decision making (Villoutreix, 2021). An ML algorithm can be defined as any model for which the model parameters, known in certain circumstances as ‘weights’ (Du and Swamy, 2014), are adjusted as the model is trained, resulting in a final version of the model that is capable of performing the specific task it was designed for. The ML field has been progressing rapidly, which naturally leads to increased interest in using these approaches for scientific research.

**Fig. 1.**
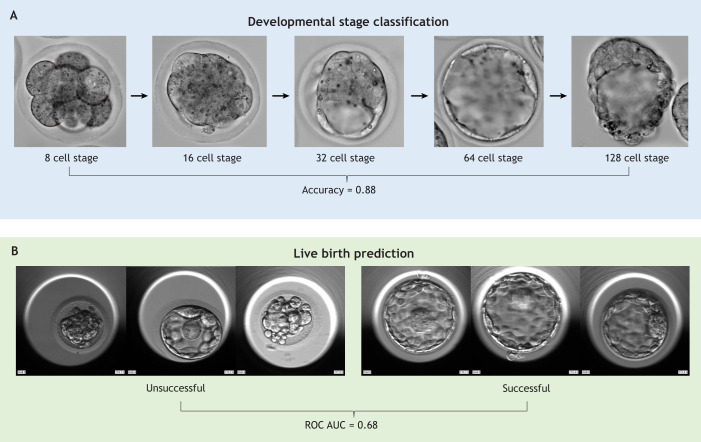
**Real life examples of classification tasks in developmental biology which can be addressed using deep learning models.** (A) Developmental stage classification performed by MobileNetV2. The model classifies developmental stage using images of mouse embryos with an accuracy of 88%. This is useful because many basic research studies first require the developmental stage of each image in the dataset to be annotated. (B) Live birth prediction performed by MobileNetV2. Using a single image at the blastocyst stage, the model predicts the probability of live birth after embryo transfer with a ROC AUC of 0.68. This tool could support IVF clinics, which need to be able to predict which embryos are likely to result in a live birth to decide which embryo(s) from a cohort to transfer. Examples taken from Mapstone et al. (2024).

In recent years, significant progress has been made to standardise the assessment of image data to allow for quicker and less subjective analyses using ML. This includes image analysis in developmental biology, for both clinical purposes such as embryo assessment in the IVF clinic (Khosravi et al., 2019; Theilgaard Lassen et al., 2023) (see Fig. 1) and for basic research purposes such as developmental staging (Yuan et al., 2014; Pond et al., 2021; Jones et al., 2024), phenotyping embryonic disease models (Naert et al., 2021) and analysing stem cell-based embryo models (Guo et al., 2021). Many different types of algorithms have been used. For example, models may vary in terms of their architecture, which refers to the type and complexity of the building blocks in the model and how these interact with each other, or in their deepness, which refers to the number of building block layers in the model (Jiang, 2021).

Choosing the best ML model for the task at hand is crucial, and this choice depends on the type and availability of data and the nature of the classification problem. Here, we aim to provide an introduction to ML for developmental biologists interested in incorporating ML into their research. We place particular emphasis on supervised learning and imaging data due to their high relevance for developmental biology. In this Spotlight, we first introduce the basics of ML and give an overview of various types of algorithms, then describe some recent examples of how these techniques have been used in developmental biology research. Finally, we provide examples of some of the latest advancements in the field of ML to bring attention to potential future developments in the application of ML to developmental biology. We also detail some key concepts and resources (see Box 1) that we believe are an essential starting point.

**Box 1. Important concepts and resources in machine learning**

**Available models.** In recent years, software packages have been developed that can be used without previous computer programming skills or knowledge. This includes the Fiji plug-in for training and using a U-Net CNN (Falk et al., 2019) and ZeroCostDL4Mic (von Chamier et al., 2021), a toolbox for training and implementing a selection of common deep learning models on microscopy images (available at: https://github.com/HenriquesLab/ZeroCostDL4Mic). With some proficiency in python, an option that allows for more flexibility in model refinement is Keras (https://github.com/fchollet/keras), an open source library that is part of the Tensorflow library. Keras can be used to define, train and test various models using very few lines of code.

**Transfer learning.** A technique that allows information gained by training one model to be used in another one. It is common practice for CNNs to first be trained on ImageNet, a visual dataset with over 14 million labelled images (Deng et al., 2009), before being refined to work on the intended dataset. The idea is that the first few layers of the base network will have been trained to recognise basic features such as edges that are found in all images and the later layers will be able to use these features to detect more complex features specific to the objects in the target dataset. It is possible to download weights learnt from pre-training on ImageNet when using Keras models.

**Data pre-processing.** Before training an ML model, it is often beneficial to undergo various data pre-processing steps that can optimise model performance. This includes standardisation, segmentation and data augmentation (Pitaloka et al., 2017; Golazad et al., 2024; Mahmud Sujon et al., 2024).

**Hyperparameter tuning.** In addition to the parameters that are learnt through the model training process, there are also fixed parameters known as hyperparameters that need to be set by the user. To achieve optimal performance, the values of these hyperparameters generally need to be tuned. For a detailed description, see Bartz-Beielstein et al. (2023).

**Interpretability.** It is often necessary to understand how models work, because this can shed light on the biological process being modelled or increase model trustworthiness (Villoutreix, 2021; Zaritsky et al., 2021; Li et al., 2022; Carvalho et al., 2019). This is often challenging with ML models, especially DNNs, because their high level of complexity makes interpretation problematic. However, although it may not always be possible to fully understand the inner workings of a model, there are techniques that can be used to try and understand which features were important to the model decision. This includes Shapley values, intuitive ablation work and the software package LIME (Ribeiro et al., 2016), which can produce explanation images where the parts of an image that were most influential in the model decision are highlighted for the user.

## Introduction to machine learning

### Supervised learning overview

**Fig. 2.**
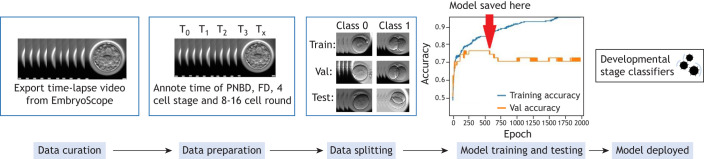
**Schema of pipeline used to develop a supervised learning model.** This pipeline shows the steps followed to develop a supervised learning model, with a human embryo developmental stage classifier used as an example (Mapstone et al., 2024). The developmental stage classifier model takes in images of an embryo (X) and outputs the developmental stage of that embryo (Y). The first step is data curation, which in this case involves exporting videos of developing human embryos from the EmbryoScope timelapse system. In the second step (data preparation) the frame numbers at relevant moments in development are annotated, and these frames are extracted from the videos. In the third step (data splitting) images from each class are assigned to the training (train), validation (val), and test sets. In the fourth step (model training and testing) the model is trained using images in the training set, and validated using images in the validation set. The version of the model that performed best for the validation set is saved. Finally, the model performance is evaluated on the test set and the model is deployed. FD, first division; PNBD, pronuclear breakdown.

Given a biological dataset, one may wish to develop a model that can make predictions or classifications from available input data. This often involves a process known as supervised learning, where a model is trained to produce an output, typically denoted Y, for a given set of input values, typically denoted X (Fergus and Chalmers, 2022) (Fig. 2). The training process uses a dataset for which the expected value of Y, often referred to as ‘ground truth’, is known. During training, the model parameters are updated in order to output a Y-value that is as close as possible to the ground truth value across all samples in the training dataset. For example, a model could be trained to predict live birth outcome (Y) after embryo transfer in an IVF procedure given a set of clinically relevant parameters (X) such as age, recognised markers of embryo quality, and infertility type, as in Goyal et al. (2020). Each individual input value is known as a ‘feature’. These features can be continuous variables, such as age and weight, or they can be categorical variables, such as type of infertility. Each input-output pair is known as a sample, so in this example a sample would be one couple with all the clinical parameters as the input and a ‘live birth’ or ‘no live birth’ label as an output.

In training, all the samples in the dataset are divided (typically by random allocation) into a training set and a validation set (Fergus and Chalmers, 2022). In some cases it is important to ensure related samples (e.g. embryos from the same couple) are always allocated to the same set during this process (Mapstone et al., 2024). The model parameters are adjusted over many iterations using training data to minimise the difference between the ground-truth values and the predicted outputs (Jiang, 2021). For each training attempt, the model performance is evaluated by calculating performance metrics for that model on the validation set. These performance metrics can then be compared to inform decisions on model type, architecture and hyperparameter values (e.g. learning rate and number of training iterations; see Box 1) so that the best model framework for the task can be identified (Jiang, 2021). Ideally, this final model would then be tested on a separate test set to determine how well it is likely to generalise to unseen data (Fergus and Chalmers, 2022); however, this step is sometimes forgone owing to data availability limitations. When training and testing a model, only labelled samples are used; once the model is developed, it is then used to predict the output label for samples where the output is not known (i.e. X is available but ground truth values of Y are not), for example predicting the chance a couple will get a live birth before a transfer to inform clinical decisions, such as how many and which embryos to transfer (Fergus and Chalmers, 2022; Goyal et al., 2020).

It should be noted that training a model successfully is heavily dependent on the quality of the data used for training. In some cases, the ‘ground truth’ labels may not always be accurate or may be prone to subjectivity. For example, if the label is the viability grade assigned to an embryo based on morphology, it is possible it might vary depending on the embryologist carrying out the assessment (Bormann et al., 2020). It is also possible that the values of the input features could be incorrect due to measurement or recording errors. In the case of a high level of errors and/or subjectivity in the feature and label values, it may not be possible to obtain a high model performance.

**Fig. 3.**
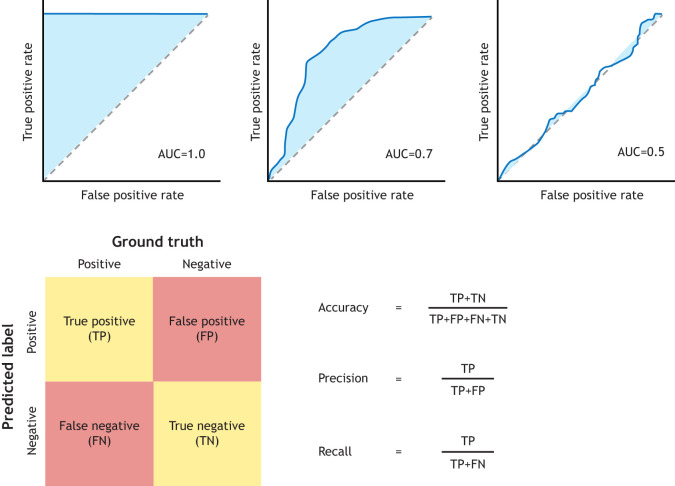
**Metrics for assessing model performance.** Top: ROC AUC curves for models with varying performances. On the left is a ROC AUC curve for a perfect model with AUC=1.0. In the middle is a ROC AUC curve for a model performing better than chance with an AUC=0.7. On the right is a ROC AUC curve for a model performing no better than chance with an AUC=0.5. Bottom: illustration of types of model predictions and how to calculate performance metrics. On the left is a confusion matrix, which defines different types of predictions depending on the combination of the ground truth and predicted label. On the right, the numbers of each type of prediction are used to calculate the accuracy, precision, and recall metrics.

There are many metrics that can be used to evaluate model performance (Fig. 3). The simplest is accuracy, which is just the proportion of samples that were correctly classified (Fergus and Chalmers, 2022). However, it is common for the number of samples in each class to be unbalanced; for example, the number of failed transfers is usually higher than the number of transfers resulting in a live birth (Mapstone et al., 2024). This means that in some cases accuracy may not be a good metric, as the model can achieve a fairly high accuracy by giving the same prediction to every sample (i.e. predicting every embryo will result in a failed transfer), yet this is obviously not useful. Therefore, for unbalanced datasets, the Receiver Operating Characteristic (ROC) Area Under Curve (AUC) metric is often used instead. ROC AUC is the area under a curve (called the ROC curve) that is created by plotting true positive rate versus false positive rate at various thresholds (see Fig. 3) (Fergus and Chalmers, 2022). The true positive rate is the proportion of positive samples that were given a positive prediction (for example, the number of correctly predicted live births divided by the total number of live births), while the false positive rate is the proportion of negative samples incorrectly classified as positive (for example, the number of failed transfers incorrectly predicted to result in a live birth divided by the total number of failed transfers) (Michelucci, 2019). A ROC AUC score of 0.5 is no better than chance, while a score of 1 represents a perfect model that will always predict the correct outcome (Fig. 3).

In general, it is good practice to use more than one metric to evaluate model performance, as this allows a better understanding of overall model performance and the type of misclassifications the model is most prone to. The best choice of metrics depends on the relative importance of different types of errors. If it is crucial to avoid false positives, for example when training a model to detect a particular cell type for further analysis, then a useful metric is precision, which assesses the proportion of positive predictions that are actually positive (Fergus and Chalmers, 2022). Alternatively, if it is more important to avoid false negatives, for example when detecting very high risk diseases, then a more useful metric might be recall, which reports the proportion of positive samples that were classified as positive (Fergus and Chalmers, 2022).

It is also important to question very high accuracy scores, as it is not unusual for a model to perform much better on the test set than when it is used in practice. This can be due to a phenomenon known as data leakage (Kauffman et al., 2011), which is when related samples end up split between the training and test set, resulting in the ability of the model to generalise being overestimated because it may have learnt to recognise specific features relevant to the related samples. Alternatively, it is also possible that the test set is not representative of the full distribution of data that the model will encounter after deployment.

### Different types of ML algorithms

Although deep learning algorithms with complex architecture and many layers are currently gaining attention, simpler ML methods have been around for decades. Common algorithms include linear regression, logistic regression, support vector machines, decision trees and random forests (see Singh, 2022). Conventional methods are less computationally expensive and often do just as well or sometimes even better than more complex methods (Russo et al., 2018), as they are less prone to a phenomenon known as overfitting, which is when a model performs very well on training data but does not generalise well. Overfitting is a common issue encountered when training ML models, especially when the number of samples is small relative to the number of features (Fergus and Chalmers, 2022). It can be detected by comparing performance metrics for the training set versus the validation or test set – if the training set performance is much higher, then this indicates overfitting (Cerulli, 2023; Fergus and Chalmers, 2022). Deep learning algorithms are particularly prone to overfitting because they typically contain millions of parameters. These parameters are capable of memorising, and therefore essentially storing, entire training datasets (Fergus and Chalmers, 2022).

Nevertheless, due to the complexity of many biological datasets (such as cases where output values depend on specific combinations of feature values), sometimes more advanced models are needed for accurate predictions (Villoutreix, 2021). This is where artificial neural networks (NNs) can be of use. The NN is an ML algorithm inspired by the biological concept of neurons. A standard supervised NN takes a list of input variables (such as mRNA and protein expression levels, developmental stage or annotated morphological features), and runs these numbers through a series of functions, or ‘hidden layers’ (LeCun et al., 2015). In a hidden layer, various combinations of variables can be constructed, allowing the model to learn complex relationships between input and output variables (LeCun et al., 2015). The outputs of each layer become the input of the next, allowing increasingly complex combinations of input variables to be engineered, resulting in new features to be fed into the final function to calculate the predicted output. There is a large amount of variety in the architecture of an NN, with different model architectures serving different needs (Jiang, 2021). For example, an NN with more than one hidden layer is referred to as a deep neural network (DNN) (Jiang, 2021). These can be useful for extracting higher level abstractions from the data (for example, shapes or edges in image data), which can be useful in complicated datasets with a large number of related features that would have otherwise needed a feature engineering pre-processing step (LeCun et al., 2015) (see Box 1).

The NN approach can be useful for analysing and classifying image data (Villoutreix, 2021). The input features for an image are the individual pixels. Each pixel is not very informative by itself, so the meaning of an image is determined by combining the feature values with their neighbouring feature values. This means that deep learning approaches are useful here. The most commonly used deep learning algorithm for image analysis is the convolutional neural network (CNN), which is specialised for image data (reviewed by Hallou et al., 2021).

Sometimes images are not stand-alone, but instead come in the form of timelapse data recordings of a biological process. In the context of developmental biology, this might be timelapse videos from live imaging of subsequent developmental stages. For this, and any other type of sequence data, a recurrent neural network (RNN) may be useful. An RNN is a type of NN that contains a looping architecture, which allows it to perform well at processing sequential data (Fergus and Chalmers, 2022). For example, Kragh and colleagues (Kragh et al., 2019) used an RNN in conjunction with a CNN to analyse timelapse videos of human blastocysts with the aim of improving viability assessment to assist embryo selection before IVF transfer. Current embryo assessment methods are usually based on static images, which does not allow for consideration of the expansion and occasional collapses of the blastocyst, or of any other dynamic processes occurring around this time. Kragh and colleagues first used a CNN to extract image features from a series of frames and then fed these features into an RNN so that temporal information could also be leveraged. The model was shown to achieve a higher correlation between predicted embryo quality and implantability than embryologists (Kragh et al., 2019).

## Recent examples of deep learning applied to developmental biology

Deep learning techniques are increasingly being adopted in developmental biology for purposes ranging from clinical decision making to basic research. In this section, we describe some recent examples to illustrate various applications of deep learning in the field.

One area where there has recently been a lot of interest in applying deep learning is embryo assessment in the IVF clinic. Currently, the process of selecting the most viable embryo to transfer is typically carried out via manual examination of timelapse videos. However, this is subjective, with up to 83% variation between embryologists (Bormann et al., 2020). Therefore, a tool that can automatically assess embryo viability would be very beneficial. There have been many attempts to develop such a tool using deep learning approaches, and a recent study (Theilgaard Lassen et al., 2023) has presented the first model to rank embryos from day 2 to day 5+, allowing flexibility in transfer regime. The model is based on 3D CNNs that simultaneously assess both morphological and temporal information from the timelapse videos.

In addition to the direct development of embryo assessment tools, deep learning can also be used for a more general investigation into the pre-implantation period. A study by Mapstone et al. (2024) identified specific moments in human pre-implantation embryo development that are most informative of embryo viability by training a CNN to predict live birth at various time-points across development. This work also demonstrated that predictions from early stages could further refine selection at the blastocyst stage, providing a valuable insight for both manual assessment methods and the development of future automated embryo assessment tools.

CNNs can also be instrumental to image analyses within basic developmental biology research. For example, Čapek et al. (2023) used a CNN approach for classifying zebrafish embryo phenotypes associated with signalling defects. The automated phenotyping tool they developed, EmbryoNet, outperformed human assessment in terms of speed, accuracy and sensitivity. Additionally, they showed that it was possible to retrain EmbryoNet to assess other fish species, demonstrating the tool has the potential to be applied to a broad range of phenomic data.

Another area of developmental biology research where deep learning has the potential to prove useful is in classifying specimens according to developmental stage, which is a crucial part of many studies. For example, a CNN can be used to classify images of chick brains into the precise sub-stages of development (Groves et al., 2023). Although a well-defined staging system exists for the embryonic chick brain, automated and precise tools like the model developed in the study are necessary to study rapid changes in gene expression that occur within a developmental stage, because the accompanying morphological changes are very subtle. Another example is KimmelNet, a CNN-based model developed for staging zebrafish embryos that was shown to give accurate predictions across a wide range of time-points (4.5 to 51 h post-fertilisation) (Jones et al., 2024). Staging of zebrafish embryos is important because many studies have reported a ‘developmental delay‘ in drug-induced or genetic phenotypes and in embryos exposed to environmental toxins (Giraldez et al., 2005; Akthar et al., 2019; Byrnes et al., 2018; Jia et al., 2020; Elabd et al., 2019; Farooq et al., 2019). Quantifying this delay can be challenging as it often requires assessing the developmental stage of large numbers of embryos, therefore automated tools such as KimmelNet could prove to be a very important resource for zebrafish research.

The majority of studies that have applied deep learning to developmental biology have followed a supervised approach; however, a recent study has demonstrated the potential of unsupervised learning (training models to identify patterns/clusters in data rather than predict output values matching ground truth values) in this field (Toulany et al., 2023). The authors used a CNN-based architecture to extract high level features to represent an image, and then calculated similarities between these features to compare images. This unsupervised approach was shown to be capable of accurately staging zebrafish embryos, and could take account of the smooth transitions between developmental stages. Therefore, this model offers an alternative to the typical approach to developmental staging that uses static idealised images of each stage based on sharp boundaries which can be difficult to define objectively. Additionally, they showed the model was capable of detecting drug-induced embryonic phenotypes in an unbiased manner, even when only normally developing embryos were used to train the model.

## Future perspectives

The field of ML is rapidly evolving, with new model architectures constantly being developed. In recent years there has been a lot of interest in transformer models (Vaswani et al., 2023 preprint), which form the basis of large language models such as ChatGPT. Transformers are a type of deep learning model that use an ‘attention‘ mechanism where significance is assigned to each word in a sentence, thus allowing context to be captured (Bahdanau et al., 2014 preprint; Kim et al., 2017 preprint). This type of model can be very useful for analysing the biomedical literature, as the vast volume of publications is a significant challenge to manual knowledge extraction and curation. For example, a recent study demonstrated that a large language model based tool, fieldSHIFT, is capable of generating testable hypotheses in the field of developmental bioelectricity (O'Brien et al., 2024).

Although originally designed for language, the transformer architecture has since been adapted for image analysis (Dosovitskiy et al., 2021 preprint). Vision transformers (ViTs) split images into patches and then use the attention mechanism to learn spatial relationships between these patches, inherently capturing global context. ViTs typically require larger datasets and more computational power than CNNs, and there is currently less established knowledge for their applications (Matsoukas et al., 2021 preprint; Takahashi et al., 2024). However, the architecture of the ViT allows better understanding of the relationship between objects in an image, and in some cases ViTs have been shown to outperform CNNs in several metrics including ROC AUC and accuracy (Matsoukas et al., 2021 preprint; Uparkar et al., 2023). ViTs have also been reported to be more robust to image distortions such as permutations and obstructions (Muzammal et al., 2021 preprint). There is now a growing interest in applying ViTs to biological image analysis. For example, Pfaendler and colleagues (Pfaendler et al., 2023 preprint) adapted publicly available self-supervised ViTs to analyse high-throughput microscopy images and found that the models were able to recognise phenotypic stem cell heterogeneity. They went on to develop scDINO, a ViT that could be trained on five-channel automated microscopy data and was able to achieve excellent performance in identifying human immune cell types.

As the variety and sophistication of ML algorithms increases, the diverse set of potential applications to biological research also grows rapidly. Developmental biology can benefit greatly from the recent advances in ML as by definition the research spans over multiple stages with dynamically changing transcriptional landscapes. This creates large, complex data sets to be analysed. We therefore believe that ML will soon become an integral part of developmental biology research.
